# Anti‐Pruritic and Analgesic Effects of a Topical Formulation Containing Troxerutin: A Pilot Study

**DOI:** 10.1111/jocd.70541

**Published:** 2025-11-12

**Authors:** Ji Young Um, Min Gyu Choi, Mi‐Sun Kim, Han Bi Kim, So Yeon Lee, Seungmin Yoo, Yeeun Cho, Chulhee Na, Nam Seo Son, Jong Gu Won, Bo Young Chung, Chun Wook Park, Hye One Kim

**Affiliations:** ^1^ Department of Dermatology Hallym University Kangnam Sacred Heart Hospital Seoul Korea; ^2^ Department of Computer Science Kwangwoon University Seoul Korea; ^3^ LG Science Park R&D Center LG Household & Healthcare (LG H&H) Seoul Korea

**Keywords:** dermatology, pain, pruritus, sensitive skin, skin barrier, topical formulation, transient receptor potential 1, troxerutin

## Abstract

**Background:**

Sensitive skin is often characterized by increased sensitivity to environmental and cosmetic stimuli, leading to pruritus and pain. Current treatments remain inadequate, leaving many affected individuals in discomfort. Troxerutin, a TRPV1‐inhibiting material, has shown potential in alleviating these symptoms by improving skin barrier function.

**Objective:**

This study evaluates the efficacy of a topical formulation containing troxerutin in reducing skin sensitivity, improving hydration, and enhancing the quality of life in individuals with sensitive skin.

**Method:**

Adult participants over 20 years old with clinically diagnosed sensitive skin applied a troxerutin‐containing moisturizer twice daily for 8 weeks. The effects were assessed using transepidermal water loss (TEWL), stratum corneum hydration (SCH), Sensitive Scale‐10 (SS‐10), and Dermatology Life Quality Index (DLQI). Measurements were taken at baseline, 2, 4, and 8 weeks.

**Results:**

Twenty adult participants who were clinically diagnosed with sensitive skin were registered. Statistically significant reductions in TEWL were observed at 2 weeks (*p* = 0.047) and 4 weeks (*p* = 0.021), indicating an improvement in skin barrier function. SCH significantly increased after 2 and 4 weeks (*p* = 0.007, *p* = 0.002). SS‐10 scores improved markedly, from 64.17 ± 12.96 at baseline to 39.84 ± 17.00 at 8 weeks (*p* < 0.001), suggesting decreased skin sensitivity. The DLQI score improved from 18.60 ± 5.00 to 8.50 ± 5.10 (*p* = 0.000), highlighting enhanced quality of life. Additionally, regional facial erythema—most notably in the cheeks and nose—was significantly reduced after 4 weeks of treatment, indicating a beneficial effect on localized vascular reactivity.

**Conclusion:**

Troxerutin significantly reduces pruritus and pain in individuals with sensitive skin and enhances skin barrier function and hydration, ultimately improving overall skin comfort and patient satisfaction.

## 1 Introduction

Sensitive skin is a widespread condition characterized by burning, tingling, itching, and pain, often triggered by various environmental, chemical, or cosmetic stimuli. Individuals with sensitive skin frequently react to nonirritating stimuli, which are harmless to those with normal skin, without any identifiable pathological skin disease [1, 2]. Everyday stimuli can elicit these reactions and may be accompanied by visible changes like erythema. However, these symptoms are not explained by any specific dermatological condition [1, 2, 3]. While the prevalence varies by country, it is estimated that around 50% of people globally (60% women and 40% men) experience sensitive skin [4].

It has been suggested that these symptoms are closely linked to the overactivation of sensory neurons, particularly those associated with the transient receptor potential vanilloid 1 (TRPV1) channel [5, 6]. TRPV1 is a nonselective cation channel that plays a pivotal role in detecting noxious stimuli such as heat, UV radiation, and chemicals [2, 7]. Activation of TRPV1 leads to an influx of calcium and sodium ions, which in turn causes nerve depolarization and subsequent transmission of nociceptive signals, resulting in sensations like itching and burning [4, 5, 6, 7]. Research has indicated that individuals with sensitive skin may exhibit heightened TRPV1 sensitivity, resulting in an exaggerated response to stimuli that do not typically affect others [7, 8].

At present, no standardized treatment exists for sensitive skin. However, dermatologists often recommend barrier‐repairing formulations, gentle cleansers, and the avoidance of known irritants such as fragrances and harsh exfoliants as part of supportive management strategies. The general recommendation for managing this condition includes the use of nonirritating cleansers and moisturizers [8, 9]. Patients with sensitive skin often have impaired skin barrier function, leading to dryness and tightness [10, 11, 12]. Studies have demonstrated that moisturizers containing soothing and barrier‐enhancing ingredients can alleviate discomfort and improve skin hydration [12, 13, 14]. However, many cosmetic products can exacerbate symptoms in individuals with sensitive skin due to ingredients like fragrances, which can lead to contact dermatitis or further irritation [15]. Troxerutin, a trihydroxyethylated derivative of the bioflavonoid rutin, has shown promise in inhibiting TRPV1 and thereby reducing skin sensitivity [16, 17, 18]. This compound, commonly found in *Sophora japonica* , is known for its anti‐inflammatory, antioxidant, and vascular‐protective properties [4]. Previous studies have highlighted its ability to reduce capsaicin‐induced TRPV1 activation, suggesting its potential use in treating conditions involving excessive TRPV1 activation, such as sensitive skin. The strong TRPV1 inhibitory effect of troxerutin distinguishes it from existing treatments for sensitive skin. In clinical practice, this suggests potential as a therapeutic option for conditions driven by TRPV1 overactivation, such as atopic dermatitis and rosacea [7]. In addition to TRPV1 inhibition, troxerutin exerts potent antioxidant [19] and vascular‐protective effects [20], which may further support its therapeutic benefit in sensitive skin.

Therefore, this pilot study aims to evaluate the effectiveness of a topical formulation containing troxerutin in alleviating the symptoms of sensitive skin, such as itching and pain, while improving skin hydration and quality of life.

## 2 Methods

### 2.1 Study Participants and Materials

The study included adult patients over 20 years old and above who had sought medical attention for the primary complaint of unpleasant sensations lasting for more than 6 weeks on their faces between April 2023 and January 2024. All participants were diagnosed with sensitive skin by two board‐certified dermatologists, based on clinical evaluation. Importantly, all participants met the diagnostic criteria for sensitive skin established by the International Forum for the Study of Itch (IFSI), and each had a Sensitive Scale‐10 (SS‐10) score exceeding 50, confirming a diagnosis of definite sensitive skin.

Inclusion and exclusion criteria were established as follows:

Inclusion criteria:

1. Adults diagnosed with sensitive skin as the primary complaint, lasting more than 6 weeks.
2. Participants who voluntarily consented to participate after receiving detailed information about the study.
3. Those capable of adhering to the study protocol and attending follow‐up visits.

Exclusion criteria:

1. Individuals with active inflammatory skin conditions (e.g., atopic dermatitis, seborrheic dermatitis) or uncontrolled systemic illnesses.
2. Participants who underwent immunotherapy or corticosteroid treatment within 4 weeks before enrollment.
3. Participants using topical corticosteroids or immunosuppressants within 1 week before the study.

### 2.2 Study Design

This study was an open‐label clinical trial to evaluate the efficacy of a topical O/W emulsion formulation containing 1% troxerutin in alleviating symptoms of sensitive skin. Participants applied the moisturizer twice daily for 8 weeks, avoiding other cosmetics except for sunscreen. Clinical evaluations and participant self‐reports were conducted at baseline, 2 weeks, 4 weeks, and 8 weeks. Assessments included objective measurements of skin barrier function, hydration, and subjective symptom severity.

### 2.3 Sensitive Skin Type Classification

Participants were categorized into one of four sensitive skin subtypes based on the Baumann classification: Allergic, rosacea, acne, and stinging. Medical records and clinical photographs were collected for analysis.

### 2.4 Outcome Measures

The primary outcome measures were transepidermal water loss (TEWL) and stratum corneum hydration (SCH), assessed using a Tewameter TM300 and Corneometer CM825 (Courage & Khazaka, Germany), respectively. Secondary outcomes included subjective symptom severity, measured using the Sensitive Scale‐10 (SS‐10) and the Dermatology Life Quality Index (DLQI). Adverse events were monitored throughout the study. TEWL was measured to assess changes in skin barrier function, with lower values indicating improved barrier integrity. SCH was measured to evaluate moisture retention in the stratum corneum, where higher values suggest improved hydration. The SS‐10 measured participants' self‐reported skin sensitivity, including pain, stinging, and itching. For the analysis of extent and strength of erythema, frontal facial photos were obtained, and adjusted for brightness and white balance.

### 2.5 Digital Image Analysis of Facial Erythema

Digital images were obtained using Janus‐III [21] manufactured by PIE Inc., which employs a high‐resolution digital camera to capture the entire face. We adopted the machine learning‐based face mesh detection module of Google MediaPipe [22] to mask the relevant face area. The severity of erythema was measured using the method proposed in [23], which decomposes the skin area image into the hemoglobin and melanin components in the log color space.

### 2.6 Statistical Analysis

All statistical analyses were performed using IBM SPSS Statistics 25.0 (IBM Co., Armonk, NY, USA). Descriptive statistics were performed on the subjects' demographic information and baseline measurements before application. The results of statistical analysis were expressed as the mean ± standard deviation, and the significance level of all statistical differences was set at a *p*‐value of 0.05 or less. A paired t‐test or Wilcoxon signed ranks test was performed to compare TEWL, Skin Hydration, SS‐10, and DLQI before application of the moisturizer at the beginning of the clinical trial, 2 weeks after application, and 4 weeks after application. A significance level of *p* < 0.05 was used for all analyses.

## 3 Results

### 3.1 Patient Population and Baseline Characteristics

Twenty participants were included in the study, all of whom met the IFSI criteria for sensitive skin and had an SS‐10 score exceeding 50, confirming their diagnosis as having definite sensitive skin. The average age was 39.60 ± 13.08 years, with 3 males and 17 females. The mean baseline TEWL on the face was 11.67 ± 5.58 g/m^2^/h, while the mean baseline SCH value was 73.71 ± 18.55 A.U. The SS‐10 score at baseline averaged 63.10 ± 12.71, and the mean Dermatology Life Quality Index (DLQI) score was 18.60 ± 5.00. The participants were classified into the following sensitive skin subtypes: Rosacea (*n* = 7), Stinging (*n* = 8), Acne (*n* = 2), and Allergic (*n* = 3). No dropouts were reported during the study period (Table 1).

**TABLE 1:** Baseline characteristics of study participants (*n* = 20).

Variables	Total (*n* = 20)
Age (years)	39.6 ± 13.1
Sex, *n* (%)	
Male	3 (15.0)
Female	17 (85.0)
Sensitive skin type, *n* (%)	
Rosacea type	7 (35.0)
Stinging type	8 (40.0)
Acne type	2 (10.0)
Allergic type	3 (15.0)
Baseline TEWL (g/m^2^/h)	11.67 ± 5.58
Baseline stratum corneum hydration (A.U.)	73.71 ± 18.55
Baseline SS‐10	63.10 ± 12.71
Baseline DLQI	18.60 ± 5.00

### 3.2 Transepidermal Water Loss (TEWL) and Stratum Corneum Hydration (SCH)

**FIGURE 1:**
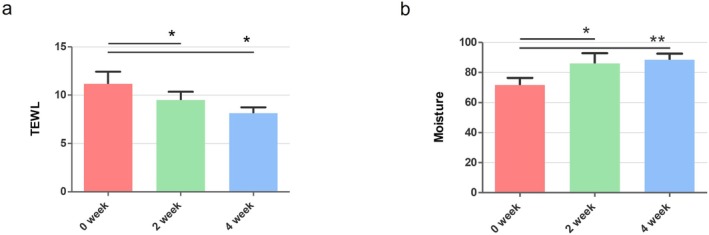
(a) Changes in TEWL at 2 weeks (Visit 2) and 4 weeks (Visit 3) compared to baseline. (b) Changes in SCH at 2 weeks and 4 weeks compared to baseline. Data are expressed as mean ± standard deviation. *p < 0.05, **p < 0.01, unit: g/m^2^/h, unit A.U.

The application of the troxerutin‐containing moisturizer significantly improved TEWL values, indicating an enhancement in skin barrier function. The mean TEWL value at baseline was 11.38 ± 5.48 g/m^2^/h. After 2 weeks of treatment (Visit 2), TEWL decreased to 8.77 ± 3.53 g/m^2^/h (*p* = 0.047), and at 4 weeks (Visit 3), it further reduced to 7.97 ± 2.20 g/m^2^/h (*p* = 0.021), both statistically significant compared to baseline (Figure 1a). This reduction in TEWL indicates that troxerutin improved the skin's barrier integrity over the course of the study.

Stratum corneum hydration (SCH) also showed a statistically significant increase. The mean SCH value at baseline was 74.75 ± 16.83 A.U. After 2 weeks, SCH increased to 88.24 ± 28.86 A.U. (*p* = 0.007), and after 4 weeks, it rose to 90.12 ± 15.52 A.U. (*p* = 0.002), demonstrating improved skin hydration over time (Figure 1b).

### 3.3 Sensitive Skin Scale‐10 (SS‐10) and DLQI (Dermatology Life Quality Index)

**FIGURE 2:**
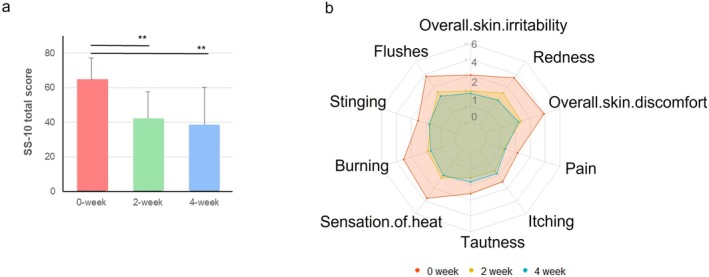
(a) Changes in SS‐10 scores at 2 weeks (Visit 2) and 4 weeks (Visit 3) compared to baseline. (b) Detailed SS‐10 symptom (overall skin irritability, redness, overall skin discomfort, sensation of heat, burning, stinging, flushes, pain, itching, and tautness) breakdown. Data presented as mean ± standard deviation. **p < 0.01.

**FIGURE 3:**
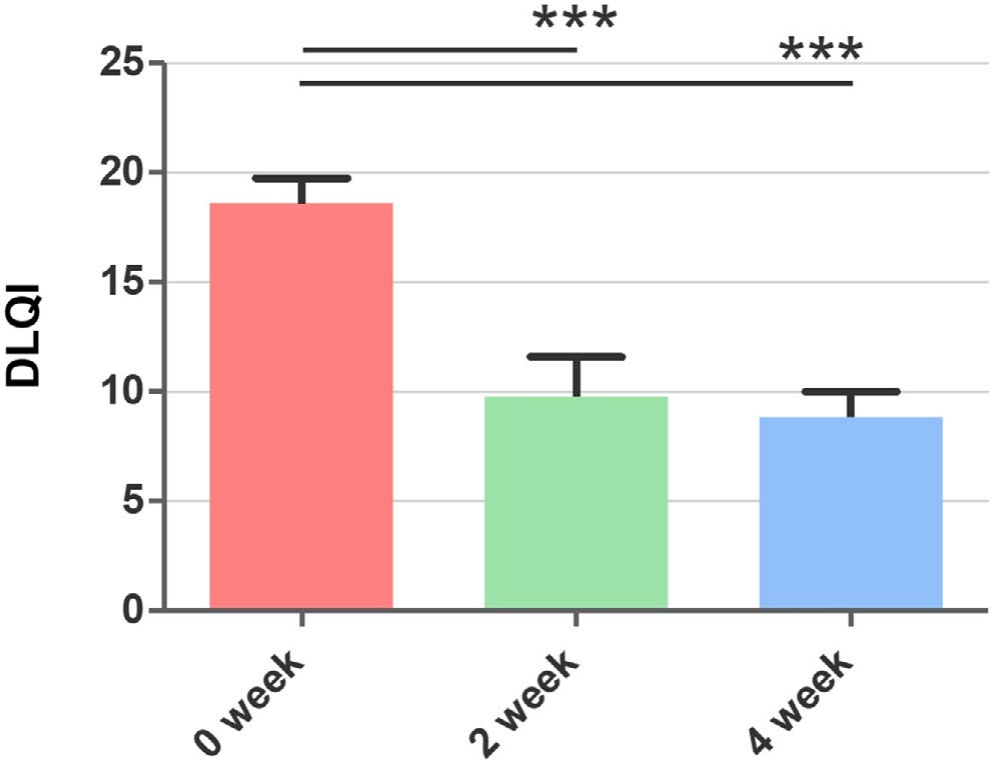
Changes in DLQI total score at 4 weeks (V3) compared to baseline. Data presented as mean ± standard deviation. ***p < 0.001.

Significant improvements were observed in the participants' subjective symptoms, as measured by the SS‐10. At baseline, the average SS‐10 score was 63.10 ± 12.71. After 2 weeks of treatment, the score improved to 52.50 ± 12.96 (*p* < 0.001), and after 4 and 8 weeks of treatment, it further decreased to 39.84 ± 17.00 (*p* < 0.001), indicating a significant reduction in subjective skin sensitivity, including symptoms such as pain, stinging, and itching (Figure 2a). In Figure 2b, a detailed analysis of SS‐10 symptoms at week 4 demonstrated statistically significant differences in overall skin irritability, redness, overall skin discomfort, sensation of heat, burning, stinging, and flushes. The DLQI score, which measures the impact of skin conditions on quality of life, also showed a substantial improvement. The mean DLQI score decreased from 18.60 ± 5.00 at baseline to 8.50 ± 5.10 after treatment (*p* = 0.000) (Figure 3). This result reflects the positive impact of troxerutin on the participants' daily lives, enhancing their overall quality of life.

### 3.4 Participant Satisfaction

**FIGURE 4:**
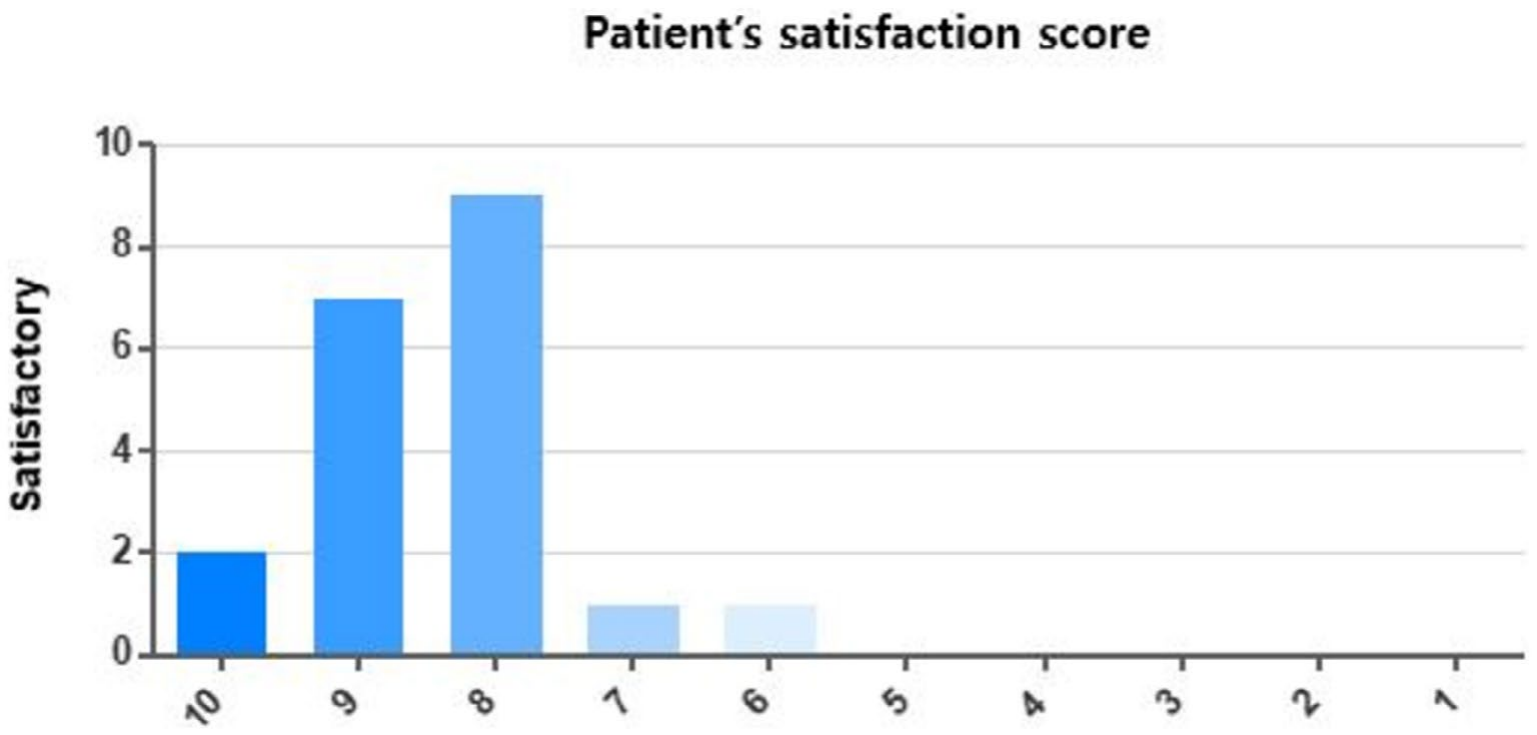
Distribution of patient satisfaction scores. Participants rated their overall satisfaction from 1 (not satisfied at all) to 10 (extremely satisfied).

Participants reported high levels of satisfaction with the troxerutin‐containing moisturizer. On a scale of 1–10, the overall satisfaction rating was as follows: 2 participants rated the product as 10/10, 6 participants rated it 9/10, 9 participants rated it 8/10, 1 participant rated it 7/10, and 1 participant rated it 6/10. Regarding the product's use and stimulation, 19 out of 20 participants indicated they would reuse the product in the future (Figure 4).

### 3.5 Regional Analysis of Facial Erythema

**FIGURE 5:**
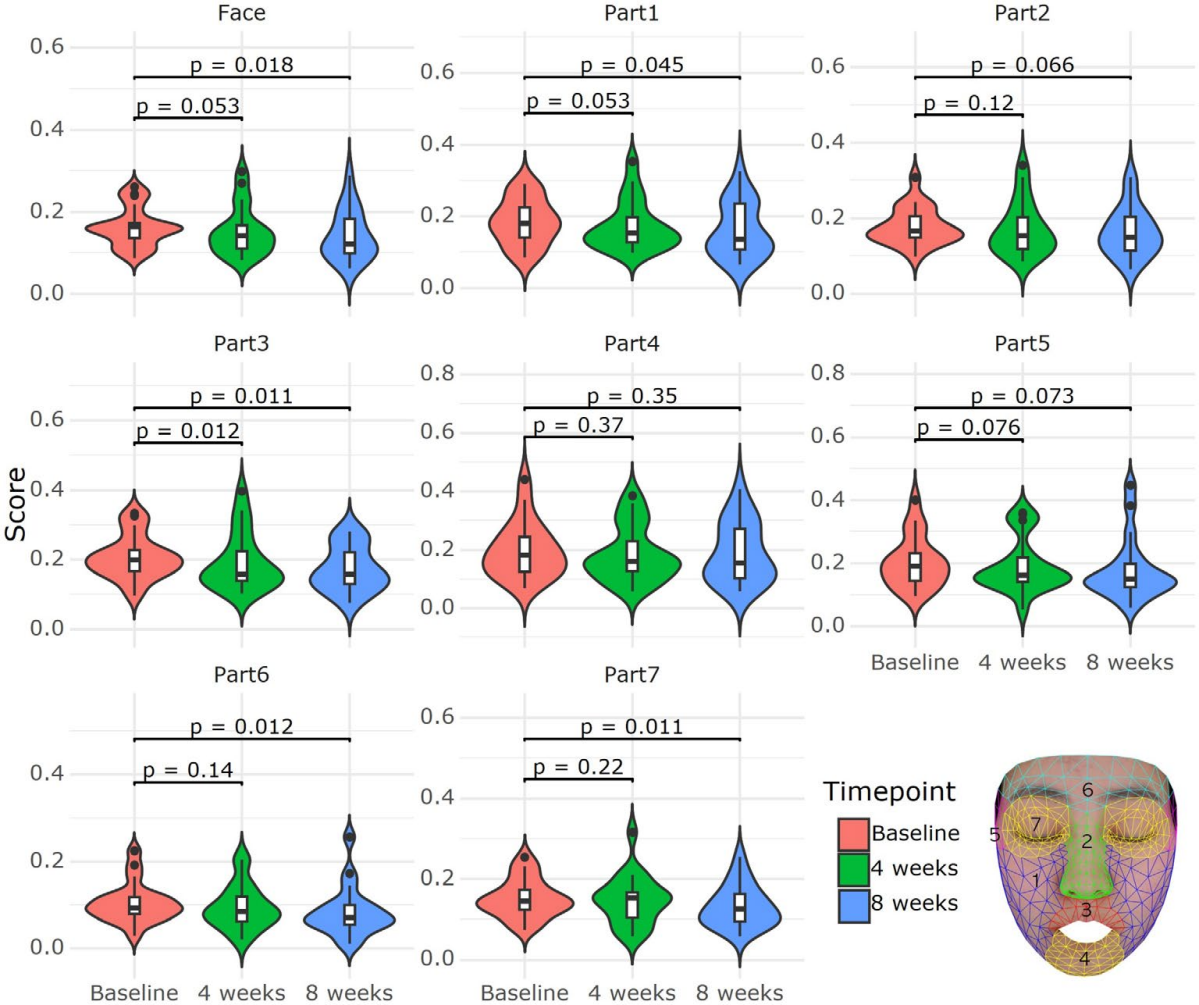
Regional distribution of facial erythema evaluated using high‐resolution digital imaging and hemoglobin decomposition analysis. The face was divided into seven anatomical regions: (1) both cheeks, (2) nose, (3) philtrum, (4) chin, (5) temporal area, (6) forehead, and (7) periorbital area. Erythema was quantified based on changes in hemoglobin signal intensity before and after application of the troxerutin‐containing moisturizer.

To evaluate the regional effects of the troxerutin‐containing formulation on facial erythema, digital facial images were analyzed across seven anatomical subregions: both cheeks, nose, philtrum, chin, temporal area, forehead, and the periorbital region. Hemoglobin signal intensity was used as a surrogate for erythema using image decomposition techniques. At baseline, the highest erythema intensity was observed in the cheeks and nose, followed by the philtrum and chin. After 4 weeks of treatment, erythema levels in these regions showed statistically significant reductions (*p* < 0.05), with the most prominent improvements noted in the cheeks and nose. Temporal and periorbital regions exhibited minimal changes due to lower baseline erythema. These findings suggest that the troxerutin‐containing formulation reduces localized vascular reactivity and improves skin redness in the most commonly affected areas of the face (Figure 5).

### 3.6 Adverse Reactions

No significant adverse reactions were reported during the study. Participants did not experience any new or worsening erythematous papules or patches, and no notable irritations were observed.

## 4 Discussion

This study demonstrated the efficacy and safety of a troxerutin‐containing topical formulation in improving skin barrier function, reducing subjective symptoms, and enhancing the quality of life for individuals with sensitive skin. All participants met the IFSI diagnostic criteria for sensitive skin, with SS‐10 scores exceeding 50, confirming their diagnosis. The results align with our initial hypothesis that troxerutin's TRPV1 inhibitory action can significantly enhance skin barrier function and reduce pruritus in sensitive skin patients, providing a promising therapeutic approach.

Troxerutin, a derivative of the bioflavonoid rutin, is known to inhibit TRPV1, a key player in skin sensitivity. In a previous study, *in silico* screening using the MT‐DTI model on natural biologically active compounds from the COCONUT database identified troxerutin as a TRPV1 antagonist [24]. This screening revealed a pharmacophore‐Tanimoto similarity score of 0.333 (maximum 1.0) when compared to the reference TRPV1 antagonist JTS‐653, marking it as a novel TRPV1 inhibitor through a chemical structure‐based drug repositioning approach [25]. This novel identification of troxerutin underscores its therapeutic potential for sensitive skin, a condition where TRPV1 overactivation plays a key role.

Furthermore, troxerutin has been shown to inhibit the mRNA and protein expression of cytokines in capsaicin‐treated keratinocytes. Clinical studies have demonstrated that troxerutin, even at low concentrations (0.1% and 0.0095%), rapidly alleviated skin erythema induced by capsaicin, a known TRPV1 activator. At higher concentrations (10%), troxerutin significantly reduced the Visual Analogue Scale (VAS) and heat sensitivity index in response to capsaicin and heat‐related stimuli [26]. These findings are consistent with the results of our study, where participants experienced a significant reduction in pruritus and skin discomfort, further supporting troxerutin's role in reducing TRPV1‐mediated hypersensitivity.

**FIGURE 6:**
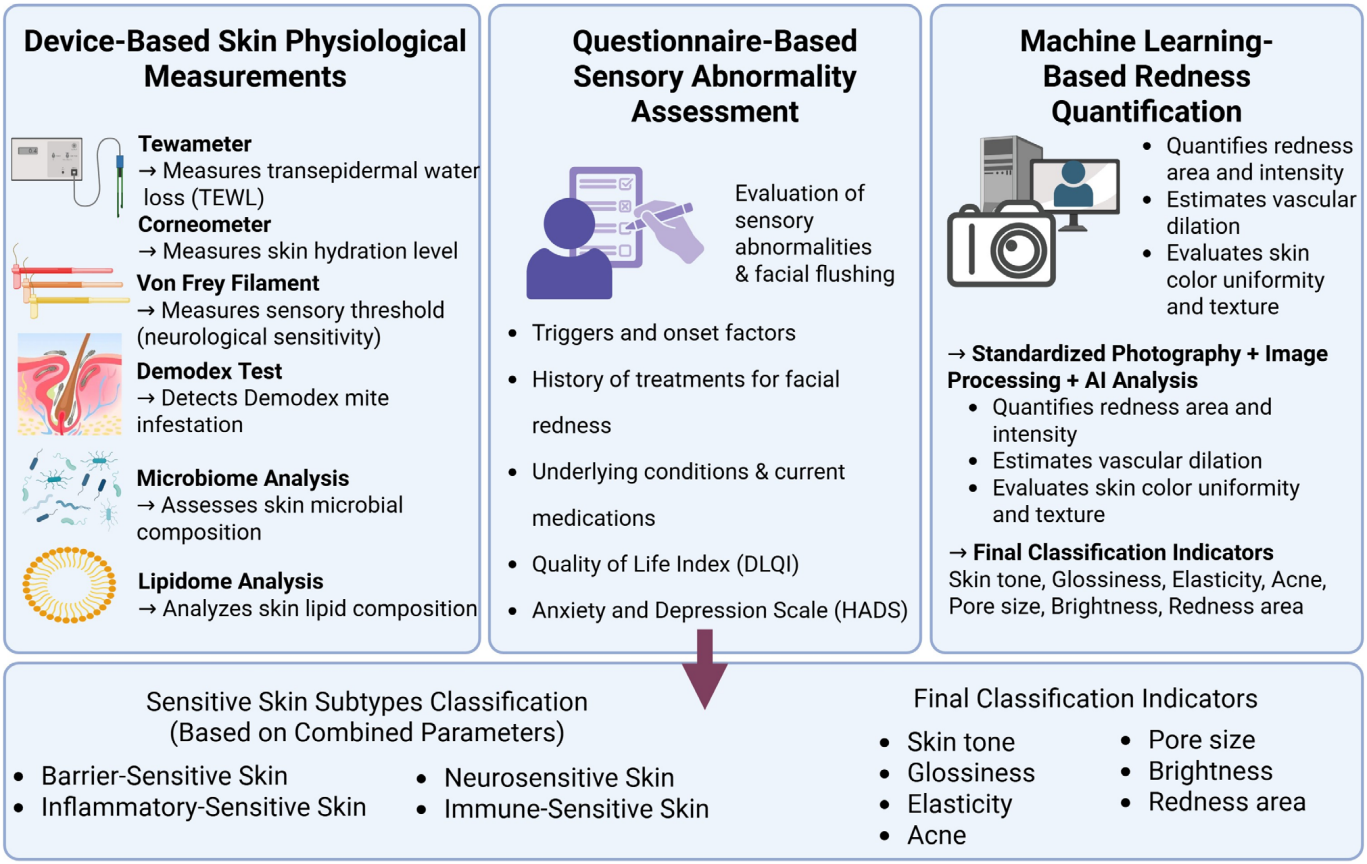
Diagnostic framework for the classification of sensitive skin. An integrated diagnostic model combining physiological measurements, questionnaire‐based assessments, and machine learning–based image analysis to classify sensitive skin subtypes. Objective measurements include transepidermal water loss (TEWL), stratum corneum hydration (SCH), Von Frey filament testing, Demodex analysis, microbiome profiling, and lipidomic analysis. Subjective assessments include symptom triggers, comorbidities, treatment history, and validated tools such as the Dermatology Life Quality Index (DLQI) and Hospital Anxiety and Depression Scale (HADS). Facial erythema and vascular reactivity are quantified through standardized photography and machine learning algorithms. Based on these data, sensitive skin is classified into barrier‐sensitive, inflammatory‐sensitive, neurosensitive, and immune‐sensitive subtypes. Additional clinical indicators such as skin tone, glossiness, elasticity, pore size, and brightness are also considered in the final classification.

The significant reduction in TEWL after 2 and 4 weeks of treatment in our study suggests that troxerutin strengthens the skin barrier, protecting it from irritants. This aligns with earlier studies demonstrating improved skin barrier function through TRPV1 inhibition [14, 15, 16, 17]. Similarly, the increase in stratum corneum hydration (SCH) highlights troxerutin's role in enhancing skin moisture, which is critical for sensitive skin prone to dryness and tightness. The ability of troxerutin to inhibit TRPV1 activation and enhance skin barrier function presents a promising therapeutic approach, not only for sensitive skin but also for conditions such as rosacea and atopic dermatitis. Compared to TRPV1 activators like capsaicin, which often induce burning sensations before desensitization [27], troxerutin offers a more tolerable profile with minimal irritation [4]. In addition, unlike flavonoids such as quercetin or hesperidin that primarily act through antioxidant pathways, troxerutin directly inhibits TRPV1, thus providing both sensory modulation and anti‐inflammatory effects [4]. In this context, another notable TRPV1 inhibitor is asivatrep, a selective agent that has been shown to effectively alleviate pruritus in patients with atopic dermatitis [28]. It is also anticipated to be beneficial in treating rosacea due to its effects on neurogenic inflammation. However, to date, its efficacy in conditions such as sensitive skin has not yet been investigated. In addition to improvements in subjective symptoms and barrier function, regional erythema analysis using digital image processing revealed a significant reduction in facial redness, particularly in the cheeks and nose which are commonly associated with high vascular reactivity in sensitive skin conditions such as rosacea. To support a more personalized approach to diagnosis and treatment, Figure 6 presents an integrated diagnostic framework combining physiological parameters, symptom‐based assessments, and machine learning–based redness quantification. This model facilitates subtype classification and enables individualized therapeutic strategies. These areas are commonly associated with high vascular reactivity in sensitive skin conditions such as rosacea. The marked improvement in erythema intensity observed after 4 weeks suggests that troxerutin may also exert beneficial effects by modulating microvascular inflammation. This objective imaging analysis further supports the clinical utility of troxerutin in treating both sensory and vascular symptoms of sensitive skin.

Despite these promising results, the study does have some limitations. The sample size was relatively small, and the study was conducted at a single center, which may limit the generalizability of the findings. The open‐label design could also introduce potential bias. Therefore, future randomized, placebo‐controlled studies are needed to validate these results and explore long‐term effects. Future research should focus on the long‐term efficacy of troxerutin and its impact across diverse populations and skin types. Comparative studies with other TRPV1 inhibitors will also be necessary to further validate its effectiveness and explore its potential in treating conditions beyond sensitive skin.

The substantial reduction in SS‐10 scores over time reflects a significant improvement in symptoms such as pain, stinging, and itching. Troxerutin's inhibition of TRPV1 effectively reduces nociceptive signals, alleviating discomfort. The improved DLQI suggests that participants experienced enhanced daily comfort and emotional well‐being, underscoring troxerutin's broader impact on quality of life. In terms of safety, no significant adverse reactions were reported. Furthermore, 95% of participants expressed a willingness to reuse the product, demonstrating its high tolerability and acceptability. In conclusion, troxerutin presents itself as a promising therapeutic agent for sensitive skin, offering significant improvements in skin barrier function, hydration, reduction of facial erythema, and overall comfort without adverse reactions. With further validation, it could become an essential option in the management of sensitive skin conditions.

Future studies should investigate the long‐term effects of troxerutin application, as well as conduct head‐to‐head comparisons with other TRPV1 inhibitors such as capsaicin or resiniferatoxin, to further elucidate its clinical positioning.

## 5 Conclusion

This study demonstrates that a topical formulation containing troxerutin is effective in reducing symptoms of sensitive skin, improving skin barrier function, and enhancing overall skin hydration. The significant improvements in TEWL, SCH, SS‐10, DLQI scores, and facial erythema support the role of troxerutin as a beneficial treatment for sensitive skin. Troxerutin's ability to inhibit TRPV1 activation, reduce localized vascular reactivity, and restore the integrity of the skin barrier makes it a valuable addition to the therapeutic options available for sensitive skin management.

Further research is warranted to explore the long‐term effects of troxerutin and its potential applications in broader populations, but these findings highlight its promising role in improving both the physical and emotional well‐being of individuals with sensitive skin. A large‐scale study is required to further validate these conclusions, underscoring the exploratory nature of this research.

## Ethics Statement

This study was approved by the Institutional Review Board (IRB no. 2023‐04‐010) at Kangnam Sacred Heart Hospital, Hallym University.

## Consent

All participants provided written informed consent prior to participation.

## Conflicts of Interest

This study was conducted as a collaboration between Hallym University Kangnam Sacred Heart Hospital, and LG Science Park R&D Center. Some authors (Seungmin Yoo, Yeeun Cho, Chulhee Na, Nam Seo Son, Jong Gu Won) are employees of LG Science Park R&D Center, while others are independent academic investigators. All authors approved the final version of the manuscript and agree with the submission.

## Data Availability

Data supporting the findings of this study are available from the corresponding author upon reasonable request.
